# Ectopic Otoconin 90 expression in triple negative breast cancer cell lines is associated with metastasis functions

**DOI:** 10.1371/journal.pone.0211737

**Published:** 2019-02-14

**Authors:** Alexander Pearlman, Mohammed Tanjimur Rahman, Kinnari Upadhyay, Johnny Loke, Harry Ostrer

**Affiliations:** Department of Pathology, Albert Einstein College of Medicine, Bronx, NY, United States of America; University of South Alabama Mitchell Cancer Institute, UNITED STATES

## Abstract

Triple negative breast cancer (TNBC) is an aggressive tumor with propensity to metastasize and poor treatment options. Improving treatment options would be impactful; thus, finding a tumor-specific cell surface protein with metastasis promoting functions that could be knocked out was the goal of this study. The Otoconin 90 gene (OC90), frequently amplified in tumors on chromosome 8q24.22, was identified as a potential therapeutic candidate. Normally OC90 is expressed in the cochlea with no known function in other normal tissues. *In silico* analysis of The Cancer Genome Atlas (TCGA) multi-tumor RNAseq cohorts revealed that OC90 is expressed in many tumor types at high prevalence and genomic amplification is associated with the elevated mRNA expression. *In vitro* assays in TNBC cell lines revealed OC90 expression with control over cell viability, apoptosis and invasion. RNA-seq analysis of OC90-siRNA knockdown and OC90-overexpression in BT20, BT549, HCC38 cell lines identified co-expressed transcripts, HMGA2, POLE2 and TRIB3. Altered expression of HMGA2, POLE2 and TRIB3 was predictive of survival among members of the Metabric breast cancer cohort. Thus, *OC90* represents a potential therapeutic target whose knockdown could improve the treatment of TNBC.

## Introduction

Breast cancer is a significant health problem, affecting up to 13% of women worldwide. It is the most common female cancer in the United States (268,670 newly diagnosed cases in 2018) and the second leading cause of cancer deaths among American women (41,400 cancer deaths in 2018) [1]. Regardless of the many advances in early detection and treatment, there is still a significant reoccurrence rate within five years of diagnosis [2–5]. One sub-type, triple negative breast cancer (TNBC), is characterized by the lack of estrogen receptor (ER), progesterone receptor (PR) and HER-2 expression [6]. TNBCs are typically high-grade and behave aggressively with a propensity to develop metastases [3]. Due to the lack of hormone receptors and HER-2 expression, these tumors are resistant to standard treatments, including hormone deprivation and Herceptin. Even toxic chemotherapies, including platinum agents, tend to be ineffective on these tumors [4]. These tumors tend to be highly aggressive with a poor long-term prognosis. Discovery of a novel anti-cancer treatment for this disease is of utmost importance for improving TNBC survival. A key feature of TNBC tumors is prevalent genomic instability with resultant chromosomal aneuploidies [6, 7]. By identifying amplified and duplicated regions within the TNBC tumor samples, potential novel anticancer targets could be identified for monoclonal antibody therapy–much as occurred with Herceptin [8]. By exploiting the genomic landscape, amplifications or deletions within primary tumors, our group developed a novel, prognostic pan cancer gene signature that predicts the metastatic potential of a variety of cancers including TNBC [9]. The Otoconin 90 gene (*OC90*), amplified on chromosome 8q24.22 was identified as one of the genes in the signature that predicted the outcome of metastatic potential.

*OC90* is normally expressed in the cochlea where it associates with Otolin and forms otoliths. It has no known protein expression in other normal tissues [10]. The *OC90* gene is amplified in breast, prostate and lung cancers, where amplification acts as an independent predictor of metastasis [9]. In that study, we demonstrated that *OC90* is representative of 154 copy number amplified genes, predictive of metastatic outcome. Of these, 56 had known metastasis functions, whereas 98 did not.

In this study we explore the effects of knockdown and overexpression of *OC90* in several TNBC cell lines of previously untested metastatic functions, including viability, apoptosis and invasion. Because the OC90 protein is normally expressed in the cochlea, behind the blood-brain barrier, the therapeutic index of knockdown by antibody or other treatment should be high (high efficacy and low toxicity). Through the use of a companion diagnostic, patients could be identified at biopsy or surgery whose tumors had amplified the target gene and should be responsive to therapy. Furthermore, by developing an ELISA test, it will be possible to demonstrate the presence of this protein in serum as an early screening marker for TNBC and as a monitoring test for response to therapy.

## Materials and methods

### Cell lines and cell culture

Human TNBC cell lines (HCC38, BT-549, HS578T, MDA-MB231, BT-20) were purchased from ATCC (Virginia, USA) maintained in DMEM/ RPMI-1640 (Life Technologies, CA, USA) supplemented with 10% fetal bovine serum (FBS), 100 U/mL penicillin, and 100 μg/mL streptomycin at 37°C jacketed with 5% CO_2_ atmosphere according to ATCC recommendations.

### Western blot analysis

Cells were harvested and lysis was performed on ice using Complete Lysis-M buffer (Roche, Mannheim, Germany) supplemented with Complete Tablets protease inhibitor cocktail. Cell lysates were boiled with Laemmli Sample Buffer at 100°C for 5 minutes. 30mg of total protein was loaded in each well of Bolt 4–12% Bis-Tris Plus (Life Tech, CA, USA) gel, run and transferred to PVDF blotting membrane (GE Health care, Freiburg, Germany) using iBolt (Life Tech, CA, USA) semi dry transfer module. Membranes were probed overnight at 4°C with primary antibodies [OC90 primary Ab (#ab110769, Abcam, MA, USA) at 1:100 dilution; GAPDH (#10R-G109a, Fitzgerald, MA, USA) at 1:10000; Caspase-3 & Caspase-7 (#9665 & #9492, Cell Signaling Technology, MA, USA) both at 1:1000] followed by respective HRP anti rabbit/anti mouse antibody incubation and developed with Pierce ECL Western Blotting Substrate (Thermo Scientific, IL, USA) on X-ray film.

### Quantitative real-time RT-PCR analysis

Relative transcript expression levels were measured by quantitative real-time PCR using a method described previously [11]. The TaqMan probe (Applied Biosystems, CA, USA) for OC90 (Cat# 4351372, ThermoFisher, MA, USA) was used to quantify the OC90 expression and GAPDH (Cat# 4331182, ThermoFisher, MA, USA) expression was used for normalizing the cDNA concentration of each sample. PCR reactions were performed in triplicate using a ViiA7 cycler (Applied Biosystems, CA, USA). Average fold changes were calculated by differences in threshold cycles (Ct) between pairs of samples to be compared. Cell lines pellet were lysed using Trizol. Total mRNA isolation was performed using an RNeasy Micro kit (Qiagen, Crawley, UK) according to the manufacturer's instructions, and RNA was eluted in 10 μl of RNase-free water. Final RNA concentration and purity were measured using a NanoDrop ND-8000 spectrophotometer (NanoDrop Technology, LabTec). Reverse transcription of 1 μg of total mRNA per sample was reverse-transcribed in 20 μL total volume using a High Capacity cDNA Reverse Transcription kit (Applied Biosystems, CA, USA) according to the manufacturer's instructions. Incubation was at 25° C for 10 min, reverse transcription was at 37° C for 120 min, and inactivation was at 85° C for 5 min. qPCR using predesigned TaqMan probes were quantified using ABI Viaa7 qPCR thermocycler, on a 384-well plates (Applied Biosystems). TaqMan Gene Expression Assay targets were predesigned; Hs00903174_m1 (OC90), Hs00971724_m1 (HMGA2), Assay Id Hs01123904_m1 (POLE2), Assay Id Hs00221754_m1 (TRIB3). GAPDH RNA was used as an internal control in the PCR reaction. 100 ng of cDNA was mixed with TaqMan Universal PCR Master Mix (Applied Biosystems). qPCR were performed in quadruplet replicates according to the manufacturer's instructions. Threshold cycle (Ct) higher than 35 as the threshold of non-expressed gene. The relative quantification (RQ) of gene expression was determined using the comparative ΔΔCt: RQ = 2-ΔΔCt with ΔCt = Ct (target gene)–Ct (endogenous gene GAPDH) and ΔΔCt = ΔCt (tumoral group) - ΔCt (normal group) in patient study or ΔΔCt = ΔCt (treated cells) - ΔCt (untreated cells) for in vitro study.

### Knockdown and overexpression of *OC90*

Knockdown of *OC90* gene expression was tested using three siRNA (Stealth RNAi, catalogue # HSS187031 (Exon 14, location: 1280), catalogue # HSS187032 Exon 14, location: 1368, and catalogue # HSS187033 Exon 5, location: 290, ThermoFisher, MA, USA) and as control siRNA, Stealth RNAi Medium GCDuplex #2 (Cat# 12935112, ThermoFisher, MA, USA) was used. Cells were seeded into 96-well plates at a density of 8000 cells per well and transfected with siRNAs using Lipofactamine. The data were plotted on a boxplot and compared to control siRNA using Student’s t test.

OC90 full genome sequence inserted into PLX304 plasmid vector was purchased from Transomic (AL, USA). Cells were transfected with OC90 expression vector and blank PLX304 vector using Lipofactamine 3000 (Cat#L3000008, ThermoFisher, MA, USA) later cells were maintained in culture media supplemented with 2μg/mL Blasticidine as selection marker.

### MTT cell viability assay

Cells were seeded into 96-well plates at a density of 8,000 cells per well and transfected with siRNAs. Cell number was determined indirectly by MTT (3-[4,5-dimethylthiazol-2-yl]-2,5-diphenyltetrazolium bromide) assay 72 hours after transfection with siRNA. Cells were incubated with 1mg/ml MTT for 2 hours, dissolved with 100μL of DMSO and read was taken with a microplate reader at 570nm. Given the significant knockdown of OC90 expression at the 48 hour time point, we thought that 72 hours would represent an optimal time point to assess phenotypic changes, such as cell viability.

### Annexin V apoptosis assay

72 hours after knockdown with *OC90*-specific siRNA, cellular apoptosis was measured using an Annexin V apoptosis detection kit (BD Biosciences, Cat# 55973) on a BD Canto II flow cytometer according to manufacturer’s instruction. The flow cytometer was calibrated for each fluorochrome and was compensated accordingly to avoid any overlap. The assay was repeated twice in sextuplet for each sample and results were analyzed using FlowJo program (V10). We calculated the percentage of apoptotic cells by combining early and late apoptotic cells percentages (based on the idea that once the early apoptotic cells are gone, they are not recounted). Each of the cell lines knocked down with OC90 siRNA was compared to their respective siRNA-treated controls to assess the significance of cellular apoptosis changes. Each run included 3 independent samples for which means were computed. All experiments were repeated at least twice. The data were plotted on a boxplot and compared to control siRNA using Student’s t test.

### Matrigel cell invasion assay

Cells were transfected 24 hours prior to seeding into Boyden chamber coated with Matrigel at 30,000 cells per chamber were placed inside. After 72 hours membranes were cut and stained with crystal violet as previously reported [12]. Each experiment were done with 3 replicates and repeated twice. The observations made on siRNA treated cell lines reflect the population of vital cells that resisted apoptosis.

### Immunohistochemistry

Cells were trypsinized, washed with PBS and fixed with 4% paraformaldehyde before suspended into pre-warmed liquefied HistoGel (Thermo Fisher Scientific, Cat#HG-4000-012) and put into a plastic mold. Later the solidified gel was transferred to a receiver paraffin block and cut into 10μm sections. The immune histochemical stain was done according to previous report with primary Anti OC90 antibody (Santa Cruz Biotechnology, Cat#sc-376744) was used at a dilution of 1:100 [13].

### Statistical significance testing

Welch 2-sample t-test was used to assess the statistical significance of Matrigel invasion, overexpression and OC90 frequency distributions in RNAseq data of TCGA from GTEx.

### RNA-seq analysis

Sequencing reads were trimmed of barcodes and aligned to the human genome (hg19) with STAR (RNA-seq aligner). Gene counts were determined with htseq-count and differential expression was measured using DESeq2 and GFOLD. Overlaps were determined using vent. Enriched functional annotations among genes were determined with Ingenuity Pathway Analysis (IPA). qPCR experiments were repeated at least three times with independent biological samples.

## Results

### *OC90* knockdown in TNBC cell lines reduces cellular viability

**Fig 1:**
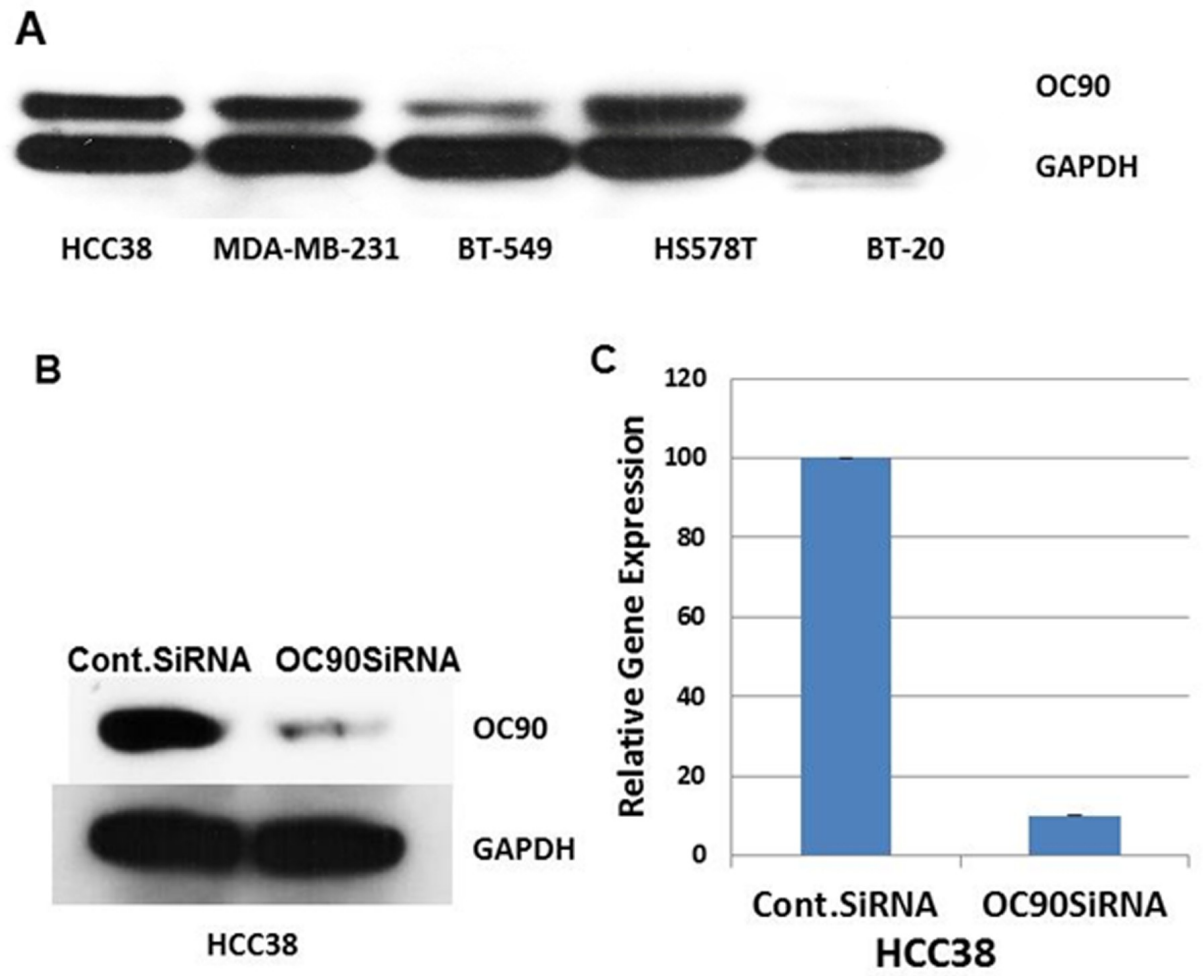
OC90 is expressed in TNBC cell lines and knocked down by siRNA treatment. A) Western blot basal expression of OC90 in TNBC cell lines relative to housekeeping protein GAPDH. B) & C) Western blot and real time RT-PCR showing mRNA and protein expression in the HCC38 cell line following OC90 specific siRNA treatment.

A panel of TNBC cell lines including HCC38, MDA-MB-231, BT-549, HS578T and BT-20 was selected to study the role of *OC90* in TNBC. Basal expression level of OC90 protein in these cell lines showed HCC38, MDA-MB-231 and HS578T have higher expression relative to low expressing BT-549 and least expressing BT-20 showed no OC90 expression (Fig 1A). OC90-specific siRNA treatment with one siRNA significantly reduced OC90 expression in the HCC38 cell line at both the mRNA and protein levels (Fig 1B and 1C). Similar efficiencies of knockdown results were observed for two other OC90 siRNAs (data not shown) and, thus, all the functional assays were performed using a single siRNA sequence to minimize off-target effects and to avoid saturating the RNAi resources. Similarly, knockdown with siRNA of HS578T and BT-549 cells was observed (data not shown).

**Fig 2:**
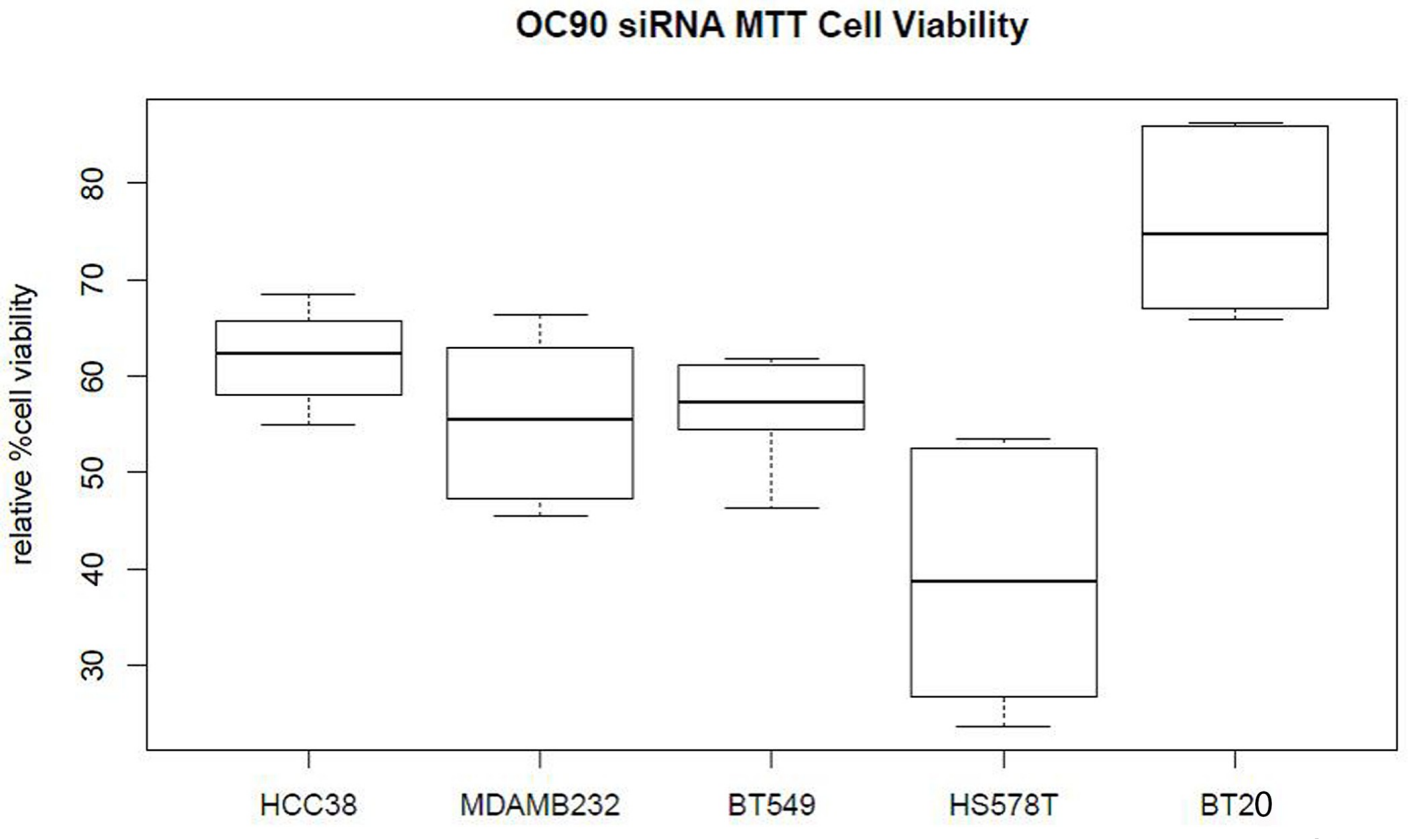
OC90 knockdown reduces cell viability in some of TNBC cell lines. Box plot shows the TNBC cell lines viability compared to that of control siRNA group.

OC90 siRNA knockdown significantly reduced cell viability in TNBC cell lines, which was more pronounced in higher expressing HCC38, MDA-MB-231 and HS578T cell lines compared to low expressing BT-549 and least expressing BT-20, as determined by MTT assay (Fig 2). Time course of the MTT assay for cell viability showed the effect was most pronounced starting at 48 hours (S1 Fig).

### Effect of *OC90* knockdown on apoptosis

**Fig 3:**
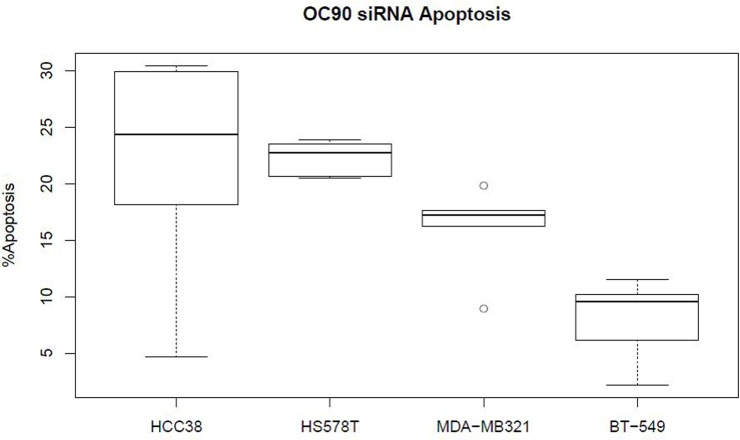
*OC90* knockdown increases cellular apoptosis in some TNBC cell lines. Box plots shows relative number of apoptotic cells (X-axis) 72 hours after *OC90* knockdown compared to vector control SiRNA in respective TNBC cell lines (x-labels). The Y-axis %Apoptosis represents changes in apoptotic cell % compared to the control siRNA group.

*OC90* knockdown significantly increased cellular apoptosis in TNBC cell lines in high expression HCC38 (P = 0.041), MDA-MB-231 (P = 0.009) and HS578T (P = 0.002) compared to low expressing BT-549 (Fig 3). Apoptosis of TNBC cell lines stimulated by OC90 knockdown was proportional to the basal expression of OC90 protein.

**Fig 4:**
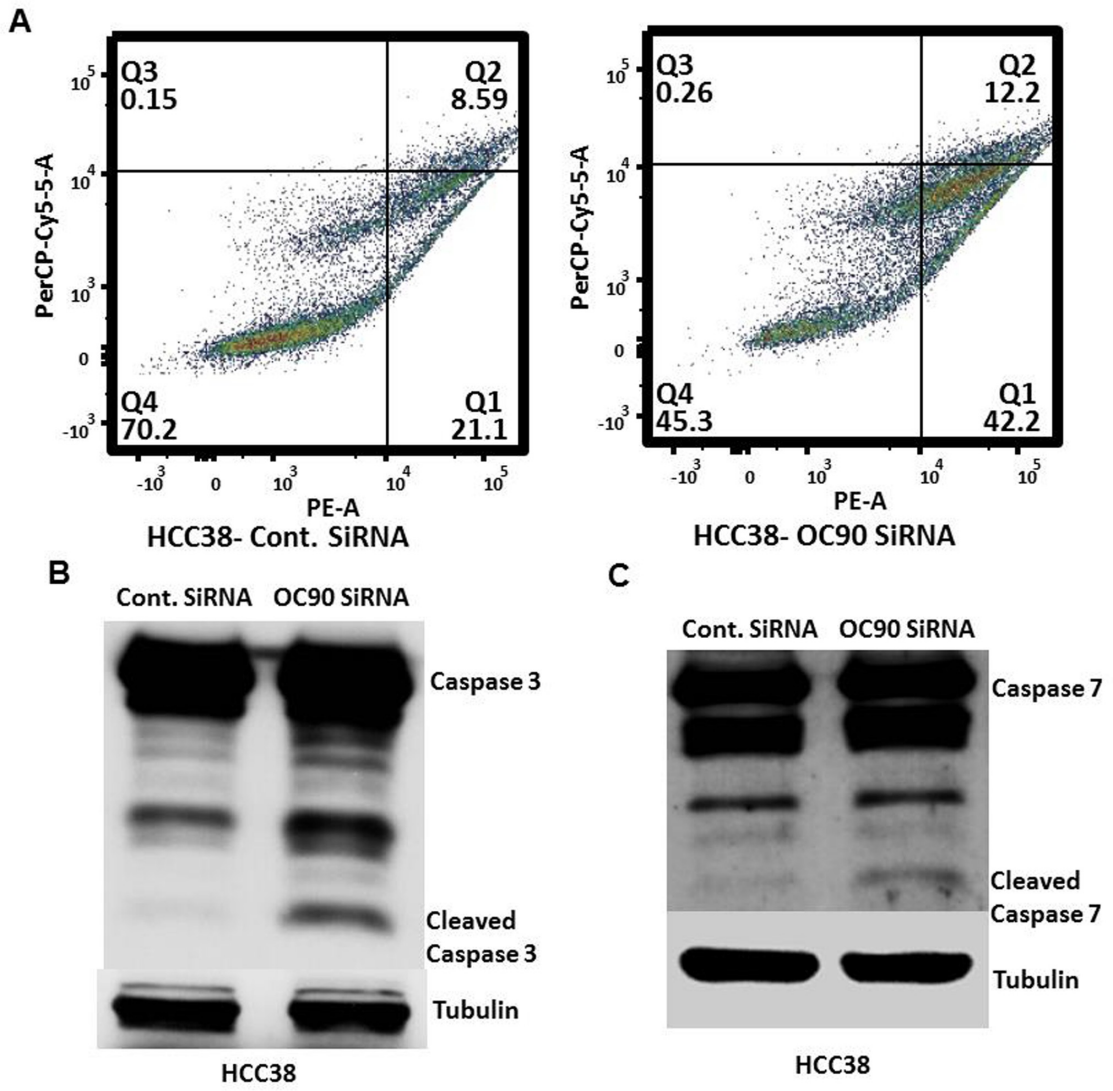
OC90 siRNA treatment promotes apoptosis in HCC38 cells. A) Flow cytometry graphs showing distribution of HCC38 cells based on Annexin V (PE, X-axis) and 7-AAD (PerCP-Cy5.5, Y-axis) intensity 72 hours after control siRNA or OC90 siRNA treatment. B) & C) Western blot showing increase in cleaved Caspase 3 & 7 (respectively) in HCC38 cell line after OC90 knockdown. Based on these findings, significant reduction in cellular viability in TNBC cell lines after *OC90* knockdown may be due to its function as an anti-apoptotic gene in TNBC and may also have some role in cellular proliferation.

Drilling into OC90 siRNA-treated HCC38 cells through flow sorting of Annexin stained cells reveals a greater proportion of apoptosis relative to the OC90 vector only control-treated cells (HCC38-control siRNA quadrant 1 (Q1) = 21.1 vs. HCC38 OC90 siRNA Q1 = 42.2) (Fig 4A). Accordingly, Western blot analysis of OC90 siRNA-treated HCC38 cells shows increased cleavage of Caspase 3 and 7, indicating its role in apoptosis (Fig 4B and 4C).

### OC90 knockdown reduces cellular invasion capabilities in TNBC cell lines

**Fig 5:**
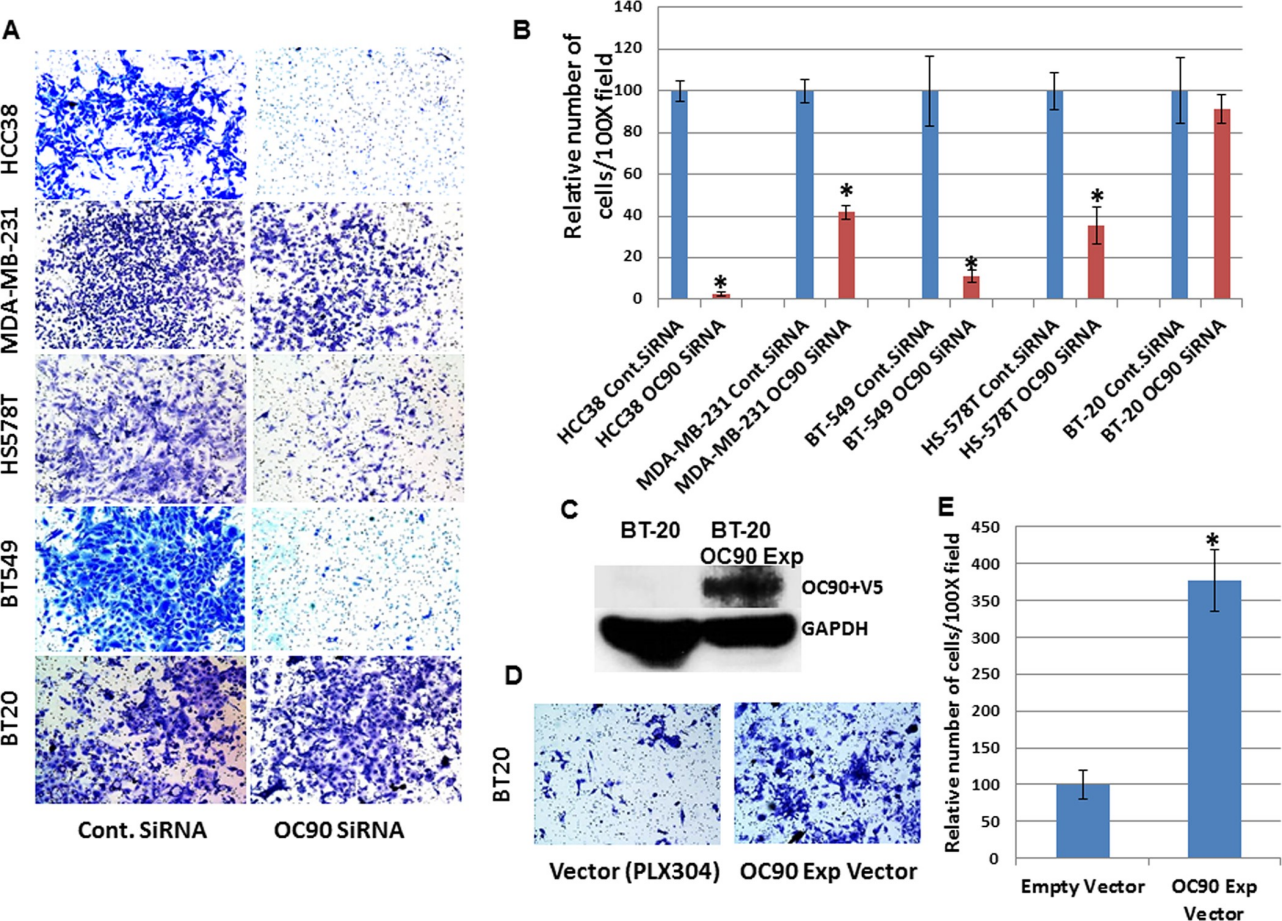
Modulating OC90 expression in TNBC cell lines affects invasion into Matrigel. A) Photographs of slides with mounted Boyden chamber membranes were counted under the high power field (100X) of the microscope following Control siRNA and OC90-siRNA treatment. B) Bar graph shows the relative number of invading cells from (A) after OC90 knockdown compared to control siRNA treatment (*p<0.05) C). Western blot showing V5 tagged OC90 expression in BT-20 cells after transfection with OC90 expression vector. D) BT-20 cell line after empty vector and OC90 expression vector transfection. E) Bar diagram shows relative number of invaded cells after empty vector and OC90 expression vector transfection in BT-20 cell line (*p < .05).

This effect was pronounced in OC90-expressing HCC38, MDA-MB-231, BT-549 and HS578T cell lines compared to very low or non-expressing BT-20 cell line (Fig 5A and 5B). On the other hand OC90-expressing vector transfection into BT-20 cell line significantly increased its invasiveness (Fig 5C–5E). All of these data suggested OC90 expression is associated with cellular invasion and metastasis in TNBC cell lines.

### *OC90* cellular localization in TNBC cell lines

**Fig 6:**
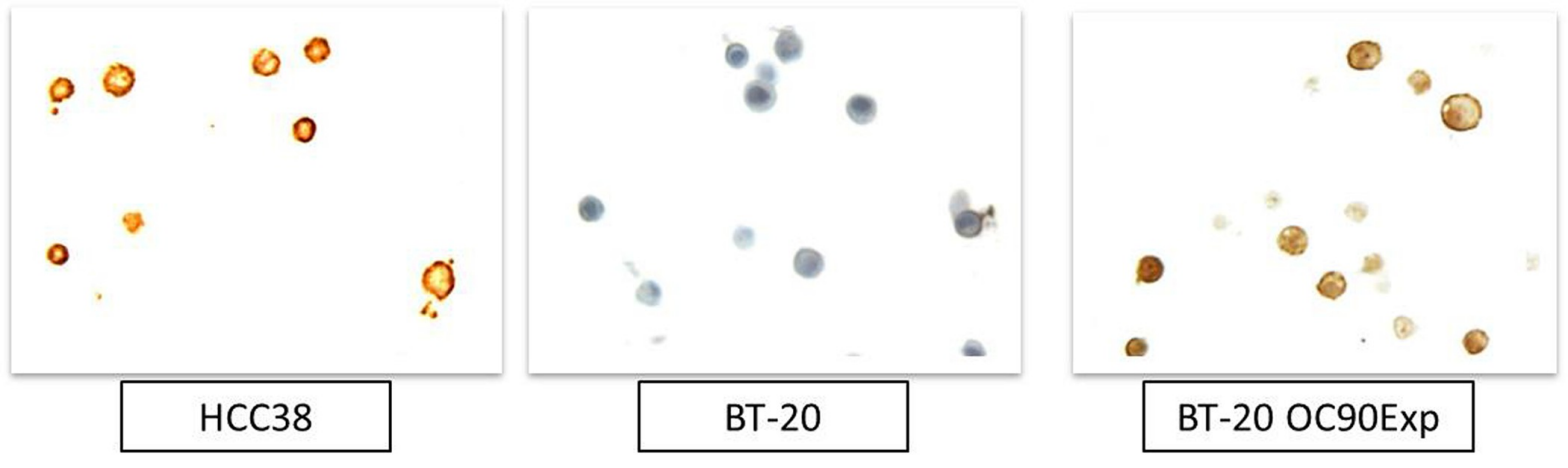
Cell membrane localization of OC90. Antibody staining of endogenous OC90 protein in HCC38 and BT-20 and BT-20 cells overexpressing OC90.

OC90 monoclonal antibody staining of HCC38 cells showed strong signal around the membrane (Fig 6). Conversely, BT20 cells that do not express OC90 protein showed no signal. BT20 cells transfected with an OC90 expression vector showed strong signal at the membrane.

### Other genes are co-expressed with OC90 when it is knocked down or overexpressed

**Fig 7:**
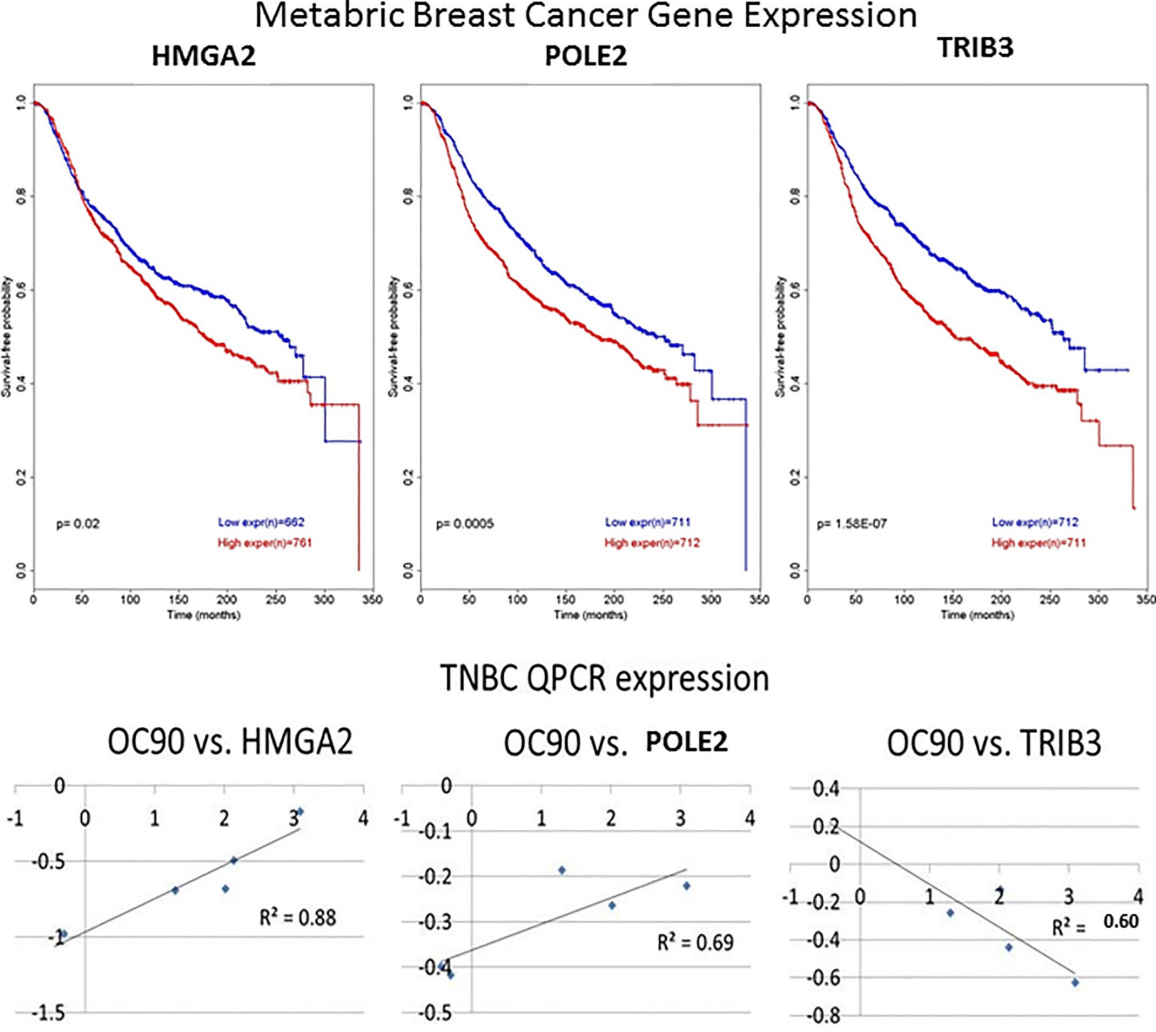
Genes demonstrating co-expression with OC90 in breast cancer predict metastasis-free survival. A) Kaplan Meier estimation of overall survival probability with *OC90* co-expressed genes, *HMGA2* (left panel), *POLE2* (middle panel), *TRIB3* (right panel). B) qPCR demonstrating co-expression with *OC90* in TNBC. Linear regression of BT20, BT549 and HCC38 under basal, knockdown and overexpression conditions (left panel OC90 vs. HMGA2, middle panel OC90 vs. POLE2, right panel OC90 vs. TRIB3).

To screen for mRNA co-expression with *OC90*, we performed RNA-seq for *OC90* basal expression, knockdown, and overexpression in three TNBC cell lines (BT20, BT549, HCC38). Of the 23 co-expressed mRNAs identified through RNA-seq, we demonstrated that overexpression of HMGA2, POLE2 and TRIB3 reduced metastasis-free survival in Metabric Breast Cancer cohort from TCGA, as observed by Kaplan Meier analysis (Fig 7A). RNAseq co-expression was demonstrated by qPCR for OC90 vs. HMGA2: r^2^ = 0.88, OC90 vs. POLE2: r^2^ = 0.69, OC90 vs. TRIB3: r^2^ = 0.60 (Fig 7B).

### *OC90* is expressed in normal and tumor tissue

Review of Genotype-Tissue Expression Project (GTEx) identified expression of *OC90* in 19.14% of normal tissues (S1 Table) [14]. Testis (82%) and various brain regions (41–73%) make up the greatest proportion of the normal OC90 expression. Likewise, expression of OC90 in tumors was observed in 19.93% of the thirty three cancer types in RNA-seq analysis of TCGA tumors by Toil (Table 1) [15]. Skin cutaneous melanoma (84%), uveal melanoma (58%) and testicular germ cell tumors (56%) have the highest proportion of OC90 expression in tumor tissues. Of note, 25% of invasive breast cancers, 20% of prostate cancers and 19% of lung adenocarcinomas show elevated *OC90* gene expression. Comparison of the gene expression magnitude as a measure of transcripts per million (TPM) between normal and tumor cohorts reveals a significant three fold increase in *OC90* expression in tumors (normal = 0.02 TPM, tumor = 0.06 TPM; p = 5.4 x 10^−9^). Furthermore the average TPM_MAX in tumors was fourteen fold greater than in normal (TPM_MAXavg tumor = 5.4; TPM_MAXavg normal = 0.38; p = 0.01), indicating a significantly wider dynamic range in tumors.

**Table 1:** OC90 RNAseq expression in TCGA tumor tissues.

Cancer Tissue	#of_samples	%OC90 expressing samples	*Max_TPM
**Skin Cutaneous Melanoma**	102	84	3.366
**Uveal Melanoma**	79	58	0.512
**Testicular Germ Cell Tumors**	148	56	13.798
**Uterine Carcinosarcoma**	57	46	0.432
**Glioblastoma multiforme**	155	34	1.25
**Pheochromocytoma and Paraganglioma**	177	33	0.377
**Thymoma**	119	29	0.566
**Kidney renal clear cell carcinoma**	542	28	1.362
**Breast invasive carcinoma**	1132	25	28.674
**Stomach adenocarcinoma**	415	24	1.168
**Uterine Corpus Endometrioid Carcinoma**	556	21	0.518
**Esophageal carcinoma**	181	20	4.172
**Kidney Chromophobe**	66	20	0.027
**Prostate adenocarcinoma**	503	20	0.077
**Lung adenocarcinoma**	537	19	9.745
**Sarcoma**	258	19	0.255
**Ovarian serous cystadenocarcinoma**	420	18	8.316
**Adrenocortical carcinoma**	77	17	0.04
**Kidney renal papillary cell carcinoma**	288	15	0.668
**Brain Lower Grade Glioma**	509	13	0.442
**Lymphoid Neoplasm Diffuse Large B-cell Lymphoma**	47	13	0.068
**Thyroid carcinoma**	504	12	0.058
**Cholangiocarcinoma**	36	11	0.017
**Lung squamous cell carcinoma**	501	10	0.879
**Cervical squamous cell carcinoma and endocervical adenocarcinoma**	304	9	0.708
**Head and Neck squamous cell carcinoma**	518	9	36.678
**Liver hepatocellular carcinoma**	369	9	0.209
**Bladder Urothelial Carcinoma**	413	8	30.239
**Mesothelioma**	87	8	0.034
**Rectum adenocarcinoma**	162	8	0.206
**Pancreatic adenocarcinoma**	178	7	0.026
**Colon adenocarcinoma**	500	6	26.589

* Max_TPM: Maximum Transcripts per Million

### *OC90* is amplified in tumor, but not normal, tissues

*OC90* copy number variation in normal tissues is a very rare event with only two samples observed to be amplified out of over 6 million samples in the current build of the Database of Genomic Variants [16]. Previously, *OC90* amplification was observed in 20–40% of TNBCs, prostate cancer and lung adenocarcinoma in our recently published analyses [9]. Similarly, 10–40% genomic DNA amplification was observed in a variety of published epithelial cancer cohorts from TCGA [17] (S2 Fig.).

### *OC90* copy number amplification is associated with an mRNA upregulation in multiple TCGA cancer cohorts

A significant increase of OC90 mRNA transcript was observed in samples with *OC90* amplification (n = 4,450) relative to tumors with copy neutral or deletions (n = 3,875) (p = 0.003) (S3 Fig). Although the connection between copy number and gene expression is complex and may be confounded by other genetic and epigenetic somatic modifications, these observations suggest a significant relationship exists for *OC90*.

## Discussion

OC90 is a soluble matrix protein that has been shown to modulate calcite crystal growth in the cochlea where it is involved in otolith formation. Otoliths function by detecting acceleration and gravity and provide a sense of stability and balance to the organism. Gene expression of OC90 mRNA was previously only observed during mouse inner ear development at days E13.5 to E15.5 [18]. Protein expression of *OC90* in other normal human tissues has not yet been observed [10]. However, more sensitive RNA-seq assay of OC90 by the Genotype-Tissue Expression (GTEx) project identified expression in approximately 20% of normal tissues [14]. Similarly, RNA-seq analysis of 33 cancer types assayed by The Cancer Genome Atlas (TCGA) also observed gene expression in approximately 20% of tumors [15]. *OC90* amplification is observed in 20–40% of TNBCs and in other cancers [9]. In this study we showed that OC90 protein expression is observed in four out of five TNBC cell lines. Overexpression is associated with increased invasion, decreased viability and apoptosis and these phenotypes can be altered by siRNA knockdown. The mechanism by which *OC90* affects viability, invasion and escape from apoptosis is not immediately apparent. This may occur from regulation of expression of other genes. Co-expression of *OC90* and *POLE2*, *HMGA2* and *TRIB3* was demonstrated in this study. Kaplan Meier analysis shows that overexpression of these genes is also predictive of decreased overall survival, but the molecular mechanism is not explained. POLE2, HMGA2 and TRIB3 genes are co-expressed with OC90 when it is knocked down or overexpressed. In previously published articles, POLE2 has been identified as part of a set of molecular biomarkers or target genes in metastatic advanced bladder cancer patients as well it is one of the genes in a model to predict survival in mantle-cell lymphoma [19, 20]. HMGA2 plays a critical role in EMT by activating the TGFβ signaling pathway, thereby inducing invasion and metastasis of human epithelial cancers [21]. Higher expression of HMGA2 has been seen in breast, ovarian and colon cancer and pancreatic adenocarcinoma [22–25]. Overexpression of TRIB3 is associated with tumor angiogenesis and a poor prognosis in patients with gastric cancer [26], and patients with high TRIB3 expression were susceptible to a recurrence of the disease, and showed poorer overall survival than those with low expression [27].

Assuming normal function of OC90 is restricted to otolith development, *OC90* has potential as a drug target, because knockdown in tumor tissues could be effective without having an impact on normal tissue function. Therefore the therapeutic index should be high. This contrasts with other overexpressed targets, such as HER2/NEU, where knockout is therapeutically effective, but because expression occurs in normal tissues and normal tyrosine kinase functions are disrupted, toxicities may also occur [28].

## Supporting information

S1 Table OC90 RNAseq expression in GTEx project normal tissues. (DOC)

S1 Fig MTT viability assay time course of TNBC cell lines. Time course (x-axis) assay of five TNBC cell lines assayed with MTT. TNBC cell lines viability (y-axis) compared to that of control siRNA group is observed with error bars representing five replicate experiments of each cell line/time point.
(DOC)

S2 Fig OC90 copy number alterations in a variety of cancer cohorts. Histograms representing frequency (y-axis) of OC90 amplification (red) or deletion (blue) in a variety of TCGA cohorts.
(DOC)

S3 Fig OC90 genomic DNA copy number effect on OC90 gene expression. RNAseq distributions of OC90 log reads (y-axis) stratified by copy number states x-axis. Welch t-test p-value is comparing diploid or deleted vs. amplified copy number states.
(DOC)

## References

[1] Siegel RL, Miller KD, Jemal A. Cancer statistics, 2018. CA Cancer J Clin. 2018;68(1):7–30. 10.3322/caac.21442 . 29313949

[2] Carey LA, Perou CM, Livasy CA, Dressler LG, Cowan D, Conway K, et al Race, breast cancer subtypes, and survival in the Carolina Breast Cancer Study. JAMA: the journal of the American Medical Association. 2006;295(21):2492–502. Epub 2006/06/08. 10.1001/jama.295.21.2492 . 16757721

[3] Clarke M, Collins R, Darby S, Davies C, Elphinstone P, Evans E, et al Effects of radiotherapy and of differences in the extent of surgery for early breast cancer on local recurrence and 15-year survival: an overview of the randomised trials. Lancet. 2005;366(9503):2087–106. Epub 2005/12/20. 10.1016/S0140-6736(05)67887-7 . 16360786

[4] Berry DA, Cronin KA, Plevritis SK, Fryback DG, Clarke L, Zelen M, et al Effect of screening and adjuvant therapy on mortality from breast cancer. The New England journal of medicine. 2005;353(17):1784–92. Epub 2005/10/28. 10.1056/NEJMoa050518 . 16251534

[5] Effects of chemotherapy and hormonal therapy for early breast cancer on recurrence and 15-year survival: an overview of the randomised trials. Lancet. 2005;365(9472):1687–717. Epub 2005/05/17. 10.1016/S0140-6736(05)66544-0 . 15894097

[6] Carey L, Winer E, Viale G, Cameron D, Gianni L. Triple-negative breast cancer: disease entity or title of convenience? Nature reviews Clinical oncology. 2010;7(12):683–92. Epub 2010/09/30. 10.1038/nrclinonc.2010.154 . 20877296

[7] Hudis CA, Gianni L. Triple-negative breast cancer: an unmet medical need. Oncologist. 2011;16 Suppl 1:1–11. Epub 2011/02/10. 10.1634/theoncologist.2011-S1-01 . 21278435

[8] Hudis CA. Trastuzumab—mechanism of action and use in clinical practice. N Engl J Med. 2007;357(1):39–51. 10.1056/NEJMra043186 . 17611206

[9] Pearlman A, Upadhyay K, Cole K, Loke J, Sun K, Fineberg S, et al Robust genomic copy number predictor of pan cancer metastasis. Genes Cancer. 2018;9(1–2):66–77. 10.18632/genesandcancer.165 29725504 PMC5931251

[10] Human Protein Atlas. Available from: https://www.proteinatlas.org.

[11] Rahman MT, Nakayama K, Rahman M, Katagiri H, Katagiri A, Ishibashi T, et al Fatty acid synthase expression associated with NAC1 is a potential therapeutic target in ovarian clear cell carcinomas. Br J Cancer. 2012;107(2):300–7. 10.1038/bjc.2012.246 22653145 PMC3394978

[12] Rahman MT, Nakayama K, Rahman M, Katagiri H, Katagiri A, Ishibashi T, et al Gene amplification of ZNF217 located at chr20q13.2 is associated with lymph node metastasis in ovarian clear cell carcinoma. Anticancer Res. 2012;32(8):3091–5. . 22843878

[13] Rahman MT, Nakayama K, Rahman M, Nakayama N, Ishikawa M, Katagiri A, et al Prognostic and therapeutic impact of the chromosome 20q13.2 ZNF217 locus amplification in ovarian clear cell carcinoma. Cancer. 2012;118(11):2846–57. 10.1002/cncr.26598 . 22139760

[14] Consortium GT. The Genotype-Tissue Expression (GTEx) project. Nat Genet. 2013;45(6):580–5. 10.1038/ng.2653 23715323 PMC4010069

[15] Vivian J, Rao AA, Nothaft FA, Ketchum C, Armstrong J, Novak A, et al Toil enables reproducible, open source, big biomedical data analyses. Nat Biotechnol. 2017;35(4):314–6. 10.1038/nbt.3772 28398314 PMC5546205

[16] MacDonald JR, Ziman R, Yuen RK, Feuk L, Scherer SW. The Database of Genomic Variants: a curated collection of structural variation in the human genome. Nucleic Acids Res. 2014;42(Database issue):D986–92. 10.1093/nar/gkt958 24174537 PMC3965079

[17] Gao J, Aksoy BA, Dogrusoz U, Dresdner G, Gross B, Sumer SO, et al Integrative analysis of complex cancer genomics and clinical profiles using the cBioPortal. Sci Signal. 2013;6(269):pl1 10.1126/scisignal.2004088 23550210 PMC4160307

[18] Deans MR, Peterson JM, Wong GW. Mammalian Otolin: a multimeric glycoprotein specific to the inner ear that interacts with otoconial matrix protein Otoconin-90 and Cerebellin-1. PLoS One. 2010;5(9):e12765 10.1371/journal.pone.0012765 20856818 PMC2939893

[19] Zekri AR, Hassan ZK, Bahnassy AA, Khaled HM, El-Rouby MN, Haggag RM, et al Differentially expressed genes in metastatic advanced Egyptian bladder cancer. Asian Pac J Cancer Prev. 2015;16(8):3543–9. . 25921176 10.7314/apjcp.2015.16.8.3543

[20] Hartmann E, Fernandez V, Moreno V, Valls J, Hernandez L, Bosch F, et al Five-gene model to predict survival in mantle-cell lymphoma using frozen or formalin-fixed, paraffin-embedded tissue. J Clin Oncol. 2008;26(30):4966–72. 10.1200/JCO.2007.12.0410 . 18606985

[21] Morishita A, Zaidi MR, Mitoro A, Sankarasharma D, Szabolcs M, Okada Y, et al HMGA2 is a driver of tumor metastasis. Cancer Res. 2013;73(14):4289–99. 10.1158/0008-5472.CAN-12-3848 23722545 PMC3715567

[22] Sun M, Gomes S, Chen P, Frankenberger CA, Sankarasharma D, Chung CH, et al RKIP and HMGA2 regulate breast tumor survival and metastasis through lysyl oxidase and syndecan-2. Oncogene. 2014;33(27):3528–37. 10.1038/onc.2013.328 23975428 PMC4096871

[23] Xi YN, Xin XY, Ye HM. Effects of HMGA2 on malignant degree, invasion, metastasis, proliferation and cellular morphology of ovarian cancer cells. Asian Pac J Trop Med. 2014;7(4):289–92. 10.1016/S1995-7645(14)60040-7 . 24507678

[24] Wang X, Liu X, Li AY, Chen L, Lai L, Lin HH, et al Overexpression of HMGA2 promotes metastasis and impacts survival of colorectal cancers. Clin Cancer Res. 2011;17(8):2570–80. 10.1158/1078-0432.CCR-10-2542 21252160 PMC3079060

[25] Piscuoglio S, Zlobec I, Pallante P, Sepe R, Esposito F, Zimmermann A, et al HMGA1 and HMGA2 protein expression correlates with advanced tumour grade and lymph node metastasis in pancreatic adenocarcinoma. Histopathology. 2012;60(3):397–404. 10.1111/j.1365-2559.2011.04121.x . 22276603

[26] Dong S, Xia J, Wang H, Sun L, Wu Z, Bin J, et al Overexpression of TRIB3 promotes angiogenesis in human gastric cancer. Oncol Rep. 2016;36(4):2339–48. 10.3892/or.2016.5017 . 27573078

[27] Miyoshi N, Ishii H, Mimori K, Takatsuno Y, Kim H, Hirose H, et al Abnormal expression of TRIB3 in colorectal cancer: a novel marker for prognosis. Br J Cancer. 2009;101(10):1664–70. 10.1038/sj.bjc.6605361 19904274 PMC2778541

[28] Montemurro F, Valabrega G, Aglietta M. Trastuzumab treatment in breast cancer. N Engl J Med. 2006;354(20):2186; author reply 10.1056/NEJMc060852 . 16707759

