# Supplementary material for: Ectopic Otoconin 90 expression in triple negative breast cancer cell lines is associated with metastasis functions
Source: PLoS One. 2019 Feb 14;14(2):e0211737. doi: 10.1371/journal.pone.0211737 (PMC6375562; doi:10.1371/journal.pone.0211737)
Supplement: S1 Table — (DOC) [file pone.0211737.s001.doc]

Ectopic OC90 Expression in Triple Negative Breast Cancer Cell Lines is Associated with Metastasis Functions.

Supporting information:

S1 Table. OC90 RNAseq expression in GTEx project normal tissues.

| **Normal Tissue** | **#of_samples** | **%OC90 expressing samples** | ***Max_TPM** |
| --- | --- | --- | --- |
| **Testis** | 165 | 82 | 0.461 |
| **Brain-Cortex** | 108 | 73 | 0.492 |
| **Brain-Hypothalamus** | 83 | 69 | 1.712 |
| **Brain-Frontal_Cortex_(BA9)** | 104 | 66 | 0.874 |
| **Brain-Substantia_nigra** | 59 | 66 | 0.807 |
| **Brain-Anterior_cingulate_cortex_(BA24)** | 84 | 63 | 1.346 |
| **Brain-Nucleus_accumbens_(basal_ganglia)** | 106 | 54 | 5.01 |
| **Pituitary** | 107 | 54 | 0.64 |
| **Brain-Amygdala** | 71 | 48 | 0.733 |
| **Brain-Caudate_(basal_ganglia)** | 112 | 46 | 1.31 |
| **Brain-Hippocampus** | 86 | 41 | 1.826 |
| **Cervix-Ectocervix** | 6 | 33 | 0.026 |
| **Adipose-Subcutaneous** | 321 | 31 | 0.619 |
| **Brain-Putamen_(basal_ganglia)** | 81 | 31 | 0.433 |
| **Brain-Cerebellum** | 121 | 28 | 0.084 |
| **Skin-Not_Sun_Exposed_(Suprapubic)** | 233 | 27 | 0.538 |
| **Cervix-Endocervix** | 4 | 25 | 0.025 |
| **Brain-Cerebellar_Hemisphere** | 98 | 20 | 0.085 |
| **Lung** | 295 | 20 | 0.123 |
| **Artery-Coronary** | 119 | 18 | 0.097 |
| **Pancreas** | 168 | 17 | 0.082 |
| **Colon-Sigmoid** | 141 | 16 | 0.067 |
| **Skin-Sun_Exposed_(Lower_leg)** | 325 | 16 | 0.147 |
| **Breast-Mammary_Tissue** | 181 | 15 | 0.455 |
| **Esophagus-Muscularis** | 246 | 15 | 0.109 |
| **Prostate** | 100 | 15 | 0.061 |
| **Kidney-Cortex** | 28 | 14 | 0.052 |
| **Brain-Spinal_cord_(cervical_c-1)** | 60 | 13 | 0.097 |
| **Artery-Aorta** | 208 | 12 | 0.082 |
| **Cells-Transformed_fibroblasts** | 260 | 12 | 0.078 |
| **Esophagus-Gastroesophageal_Junction** | 137 | 12 | 0.071 |
| **Spleen** | 100 | 12 | 0.18 |
| **Adipose-Visceral_(Omentum)** | 198 | 11 | 0.143 |
| **Bladder** | 9 | 11 | 0.031 |
| **Colon-Transverse** | 167 | 10 | 0.1 |
| **Heart-Atrial_Appendage** | 175 | 9 | 0.048 |
| **Ovary** | 88 | 9 | 0.03 |
| **Adrenal_Gland** | 128 | 8 | 0.059 |
| **Artery-Tibial** | 284 | 7 | 0.164 |
| **Cells-EBV-transformed_lymphocytes** | 107 | 7 | 0.023 |
| **Minor_Salivary_Gland** | 55 | 7 | 0.024 |
| **Muscle-Skeletal** | 397 | 7 | 0.073 |
| **Thyroid** | 283 | 7 | 0.071 |
| **Whole_Blood** | 340 | 7 | 0.056 |
| **Stomach** | 175 | 6 | 0.064 |
| **Heart-Left_Ventricle** | 205 | 5 | 0.094 |
| **Small_Intestine-Terminal_Ileum** | 93 | 5 | 0.026 |
| **Vagina** | 85 | 5 | 0.026 |
| **Esophagus-Mucosa** | 274 | 4 | 0.047 |
| **Nerve-Tibial** | 278 | 4 | 0.136 |
| **Liver** | 110 | 3 | 0.024 |
| **Uterus** | 79 | 3 | 0.061 |
| **Fallopian_Tube** | 5 | 0 | 0.000 |

* Max_TPM: Maximum Transcripts per Million

S1 Table. Gene expression assayed through RNAseq in a variety of normal tissues (column one). Column two represents the number of samples assayed and column three is the percentage of OC90 expressing samples. Column four represents the maximum transcripts per million for the specific tissue.
