# Supplementary material for: Ectopic Otoconin 90 expression in triple negative breast cancer cell lines is associated with metastasis functions
Source: PLoS One. 2019 Feb 14;14(2):e0211737. doi: 10.1371/journal.pone.0211737 (PMC6375562; doi:10.1371/journal.pone.0211737)
Supplement: S1 Fig — Time course (x-axis) assay of five TNBC cell lines assayed with MTT. TNBC cell lines viability (y-axis) compared to that of control siRNA group is observed with error bars representing five replicate experiments of each cell line/time point. (DOC) [file pone.0211737.s002.doc]

Ectopic OC90 Expression in Triple Negative Breast Cancer Cell Lines is Associated with Metastasis Functions.

Supporting information:

S1 Fig. OC90 copy number alterations in a variety of cancer cohorts.


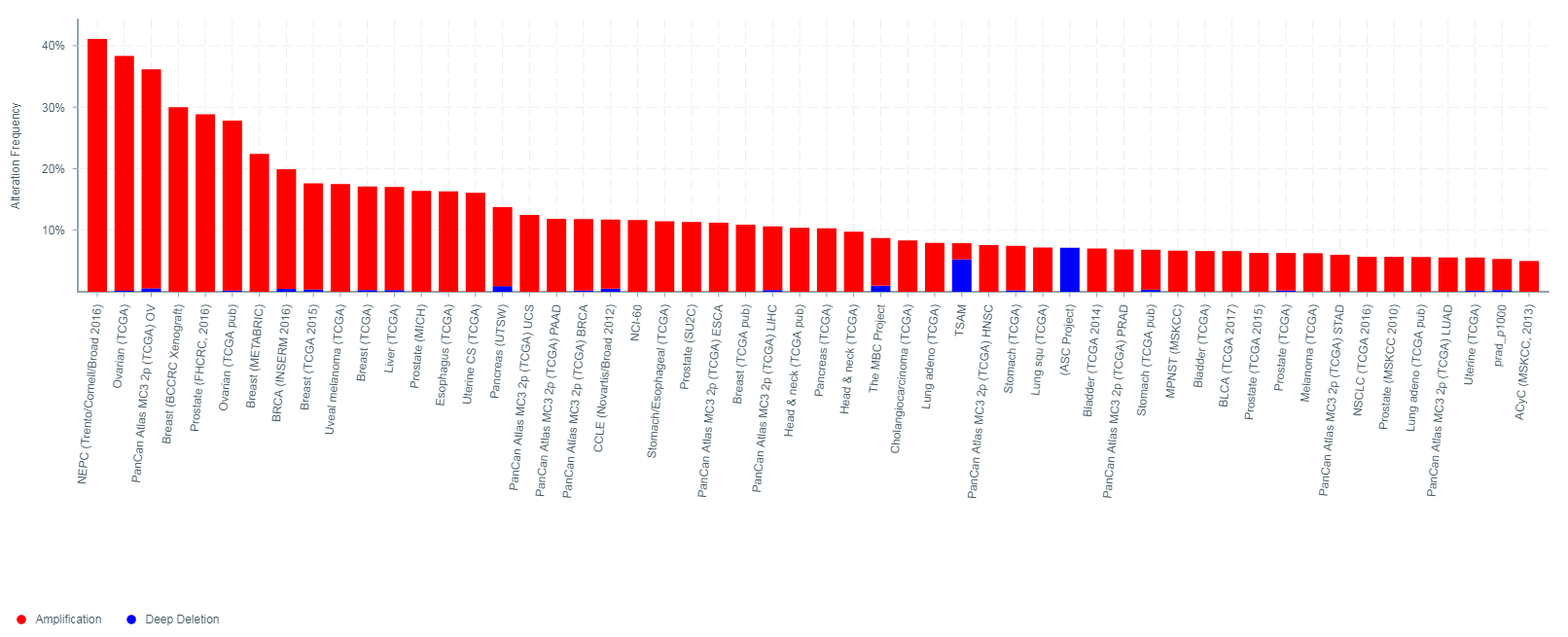


S1 Fig. Histograms representing frequency (y-axis) of OC90 amplification (red) or deletion (blue) in a variety of TCGA cohorts. Figure exported from <http://www.cbioportal.org/>
