# Supplementary material for: Ectopic Otoconin 90 expression in triple negative breast cancer cell lines is associated with metastasis functions
Source: PLoS One. 2019 Feb 14;14(2):e0211737. doi: 10.1371/journal.pone.0211737 (PMC6375562; doi:10.1371/journal.pone.0211737)
Supplement: S2 Fig — Histograms representing frequency (y-axis) of OC90 amplification (red) or deletion (blue) in a variety of TCGA cohorts. (DOC) [file pone.0211737.s003.doc]

Ectopic OC90 Expression in Triple Negative Breast Cancer Cell Lines is Associated with Metastasis Functions.

Supporting information:

S2 Fig. OC90 genomic DNA copy number effect on OC90 gene expression.

**
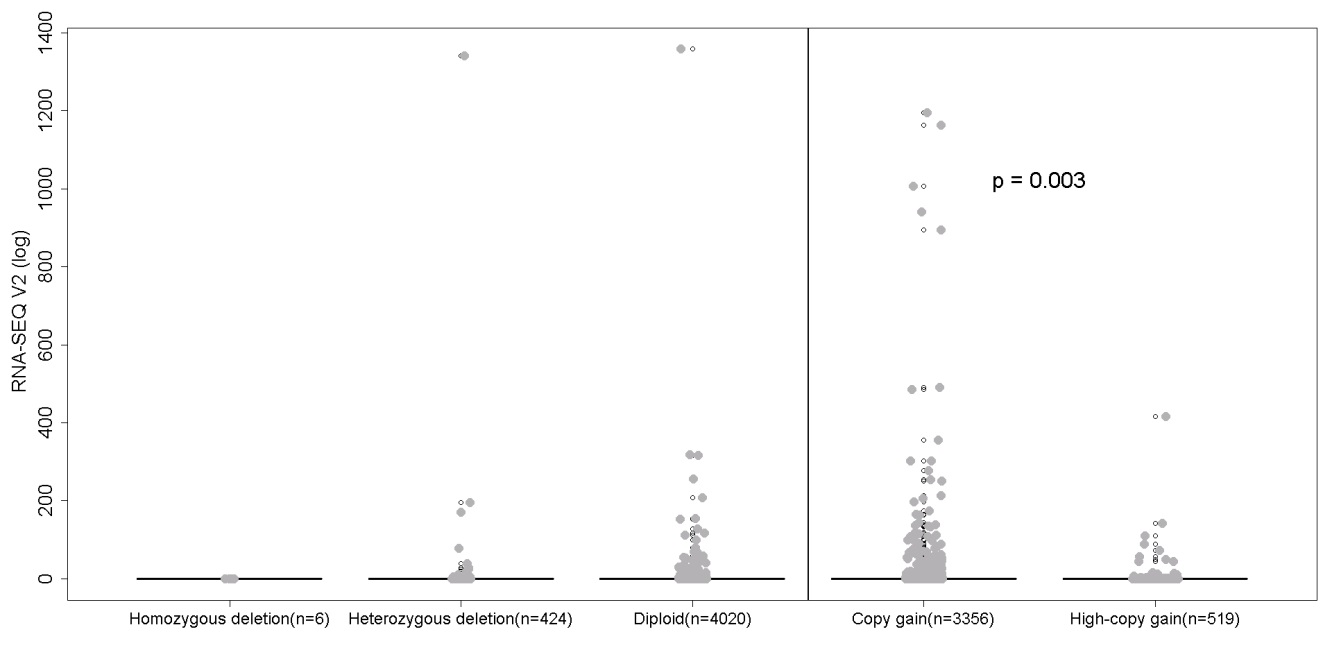
**

S2 Fig. RNAseq distributions of OC90 log reads (y-axis) stratified by copy number states x-axis. Welch t-test p-value is comparing diploid or deleted vs. amplified copy number states.
